# What Is Required for AI to Improve the Assessment and Treatment of Patients With Lower Urinary Tract Dysfunction? ICI‐RS 2025

**DOI:** 10.1002/nau.70186

**Published:** 2025-11-12

**Authors:** Glenn T. Werneburg, Michel Wyndaele, John E. Speich, Enrico Finazzi Agro, Mo Belal, Sachin Malde, George Bou Kheir, Emre Huri, Arjun K. Nambiar, Giovanni Mosiello, Riccardo Lombardo, Phil Toozs‐Hobson, Christopher R. Chapple, Alan J. Wein, Paul Abrams, Kevin Rademakers

**Affiliations:** ^1^ University of Michigan Ann Arbor Michigan USA; ^2^ Cleveland Clinic Cleveland Ohio USA; ^3^ Department of Urology University Medical Center Utrecht Utrecht The Netherlands; ^4^ Department of Mechanical and Nuclear Engineering Virginia Commonwealth University Richmond Virginia USA; ^5^ Urology Unit, Policlinico Tor Vergata University Hospital Rome Italy; ^6^ Department of Surgical Sciences University of Rome Tor Vergata Rome Italy; ^7^ Department of Urology Queen Elizabeth Hospital Birmingham UK; ^8^ Department of Urology Guy's and St. Thomas' NHS Foundation Trust London UK; ^9^ Department of Urology, Ghent University Hospital ERN accredited centrum Ghent Belgium; ^10^ Department of Urology Hacettepe Üniversitesi Ankara Turkey; ^11^ Department of Urology Freeman Hospital Newcastle‐upon‐Tyne UK; ^12^ Division of Neuro‐Urology Bambino Gesù Children”s Hospital Rome Italy; ^13^ Unit of Urology, Sant' Andrea Hospital Sapienza University Rome Italy; ^14^ Department of Gynaecology Birmingham Women's NHS Foundation Trust Birmingham UK; ^15^ Department of Urology, Sheffield Teaching Hospitals NHS Foundation Trust University of Sheffield UK; ^16^ Perelman School of Medicine, Penn Medicine University of Pennsylvania Philadelphia Pennsylvania USA; ^17^ Desai Sethi Institute of Urology, Miller School of Medicine University of Miami Miami Florida USA; ^18^ Bristol Urological Institute Bristol UK; ^19^ Department of Urology Zuyderland Medical Center Heerlen The Netherlands

**Keywords:** artificial intelligence (AI), bladder outlet obstruction (BOO), EU AI Act, lower urinary tract dysfunction (LUTD), machine learning, overactive bladder (OAB), Software as a Medical Device (SaMD)

## Abstract

**Introduction:**

Artificial intelligence (AI) is poised to improve the diagnosis and management of lower urinary tract dysfunction (LUTD). Its effective deployment requires prioritization, regulatory oversight, rigorous validation, and clinician and patient engagement.

**Methods:**

The Think Tank at the International Consultation on Incontinence—Research Society (ICI‐RS) 2025 evaluated key considerations for successful AI implementation into LUTD clinical care. The topics included clinical triage framework, regulatory and legal principles, levels of evidence required for validation, and clinician and patient engagement to guide development. The group developed a narrative of the pressing matters related to AI implementation and a list of proposed research questions, which, when addressed, will help shape the future of the field.

**Results:**

LUTD topics that should be prioritized for AI implementation include high‐burden conditions with high unmet need such as neurogenic LUTD, bladder outlet obstruction, and overactive bladder. Regulatory frameworks such as the EU AI Act and the U.S. “Software as a Medical Device” and its associated guidance promote safety, transparency, and accountability. AI solutions should be as rigorously evaluated as other clinical devices or drug agents. Patient and clinician engagement are paramount to ensure innovation aligns with the pressing needs of patients and clinicians.

**Conclusions:**

AI's integration into LUTD care requires cross‐disciplinary collaboration, prospective validation, and legal and ethical frameworks. AI must be developed and implemented with a strong focus on transparency, trust, and patient‐centered care.

**Clinical Trial Registration:** This study is not a clinical trial and thus does not warrant registration as such.

## 1 Introduction

Artificial Intelligence (AI) carries major potential for public health authorities, healthcare companies (pharmaceutical or tech), healthcare providers and professionals, and patients in general. It is on the cusp of transforming the diagnosis, management, and long‐term care for individuals with lower urinary tract dysfunction (LUTD). AI has demonstrated value throughout medical domains including imaging interpretation, risk prediction, and clinical decision support, yet its implementation in LUTD is in its infancy. In the near future, patients could receive personalized assessment and treatment, be assisted by chatbots, and benefit from healthcare apps. Healthcare professionals on the other hand could benefit from automation of repetitive tasks, ad hoc data analysis to advise on diagnosis or treatment, systematic reviews and guideline development. To ensure meaningful and sustainable adoption, AI should be deployed based on a systematic triage strategy. Priority should be placed on clinical scenarios with high unmet need, those with a significant disease burden, the need for treatment personalization, and/or resource limitations. Conditions with multiple decision points and therapeutic options such as neurogenic LUTD and overactive bladder (OAB), as well as tests with variable clinical interpretation such as bladder diaries or urodynamics, are ideally suited for AI implementation (Table 1).

**Table 1:** Applications of artificial intelligence in lower urinary tract dysfunction and their considerations.

Example application	Description	Pros	Cons
AI Interpretation of Uroflowmetry	Pattern recognition for diagnosing voiding dysfunction subtypes	Standardised interpretation, reduces interobserver variability	Requires large validated labelled datasets
AI‐Guided Pelvic Floor Therapy Apps	Tailored exercise regimens with feedback for stress or mixed incontinence	Increases accessibility, reduces physiotherapy demand, may improve adherence	Limited effectiveness without patient motivation, requires patient ability to use computer‐based technology
Chatbots for Bladder Training	AI chatbots guiding behavioural therapy and hydration habits	Enhances adherence, scalable	Risk of misinformation if unsupervised
Neurogenic upper tract risk stratification models using urodynamics and videourodynamics	Predicts deterioration in SCI and spina bifida patients or early intervention	Prevents complications, prioritises follow‐up	May requires integration with both EMR and urodynamics system, long‐term data validation
Surgical Treatment Planning	AI defining surgical treatment plan based on radiological and clinical data	Increased efficiency and precision during surgery	Requires standardisation of radiological and endoscopic imaging
Monitoring patient adherence	AI access to treatments and prescription refills, adherence to surveillance regimen in high‐risk LUTD	Real time alerts	Integration with primary care

Successful AI solutions should also be designed within robust legal, regulatory, and ethical frameworks, which together ensure data quality, patient privacy, accountability, and equitable access. The levels of evidence necessary for AI validation should meet or exceed those necessary for other diagnostic and therapeutic standards. The perspectives of both patients and clinicians should be sought early and incorporated consistently to maximize utility, trustworthiness, and practical relevance.

In this paper, which stemmed from the International Consultation on Incontinence—Research Society (ICI‐RS) 2025, we explore the current landscape and future of AI in functional urology. We review a triage framework for AI application in LUTD, the regulatory and ethical considerations necessary for AI implementation, and evaluate the levels of evidence necessary for validation. We discuss the critical role of the patient and the clinician in guiding responsible AI‐based innovation. Finally, we sought to develop high‐priority research questions and strategies to address them with a focus on AI use to advance LUTD research and patient care (Table 2).

**Table 2:** Research questions and strategies to address them.

Research question	Strategy
Can AI write a reliable systematic review on lower urinary tract dysfunction (LUTD)?	LUTD review written by AI compared to those written by healthcare professionals
Can AI develop a reliable LUTD care guideline?	NLUTD guideline written by AI compared to those written by healthcare professionals
Can patients be re‐identified from an anonymised data set?	Deidentified database of urodynamics tracings
Can AI be used for regulatory decision‐making in LUTD care?	Reimbursements, decision‐aids, supervision
Does the difference in AI regulation impact implementation of AI for LUTD care in different countries?	Developed and developing world, U.S. and EU
Can AI assist with or replace patient monitoring?	Bladder diaries, pad tests
What is the environmental burden of AI?	Is the environmental burden justified by the improvement in clinical care?
Should priority be placed on applications of clinical importance or on applications with data (structure or volume) most amenable to handling by AI?	Data set with large volume in a condition with low clinical research priority or small volume with high clinical research priority condition
How and at what stage can clinicians and patients be formally engaged in AI‐based algorithm development?	Interviews to identify unmet needs; structured interviews early in algorithm development process once application is identified
What AI applications will be covered by insurance?	Review of payor policies by region/country, analysis of reimbursement decisions
Does providing clinicians and patients with AI‐generated treatment recommendations for LUTD improve trust, shared decision‐making, and/or treatment adherence compared to recommendations without explanation?	Randomized controlled clinical trial with an explainable versus non‐explainable AI
Who will pay for AI applications? Insurance, NHS, Stakeholder (as industries)?	Market analysis, cost‐effectiveness analysis, stakeholder interviews

## 2 How Can the Open Questions in LUTD be Triaged as Potential Applications of AI?

The deployment of AI in LUTD management requires a systematic triage strategy to maximise clinical and research impact. A structured selection framework enhances the value of AI by focusing on high‐priority, data‐rich, and outcome relevant issues [1]. Clinical impact must guide application selection, targeting conditions with significant morbidity or quality‐of‐life burden such as neurogenic LUTD [2], severe bladder outlet obstruction (BOO) and posterior urethral valves (PUV) [3], or refractory OAB [4, 5]. Data availability and technical feasibility are important; AI algorithms require structured datasets such as those from uroflowmetry, bladder diaries, (video)urodynamics, and kidney and bladder ultrasound to train predictive models reliably. Variability in diagnosis identifies conditions where interpretation differs substantially among clinicians. For example, AI may standardize assessment of interpretation of bladder diaries or uroflowmetry patterns. Resource bottlenecks can direct AI towards settings with limited specialist availability. For example, virtual pelvic floor therapy guided by AI can expand access and reduce dependence on in‐person physiotherapy. Further, it may improve patient adherence. Patient engagement potential should be assessed, favouring applications that empower self‐management. Chatbots and digital adherence tools can enhance behavioural therapy, fluid management, voiding retraining, and adherence to surveillance regimens in high risk LUTD. Table 1 lists potential AI applications in LUTD and their considerations.

AI could be useful in the diagnosis of common conditions as well as complex LUTD requiring life‐long management to mitigate risk of renal failure. PUV diagnosis and management could benefit by AI. For example, AI may assist in prenatal diagnosis, selection of candidates for fetal treatment or post‐natal resection, and long‐term evaluation of bladder and renal function [6, 7]. Finally, risk stratification is a powerful AI application in LUTD, enabling early prediction of deterioration such as autonomic dysreflexia or upper tract damage in individuals with neurogenic LUTD, thus guiding timely intervention. This triage framework ensures AI resources are allocated towards clinically meaningful, technically feasible, and ethically responsible innovations, improving diagnostic precision, personalising treatment, and optimising health system efficiency [8].

## 3 What Are the Legal, Regulatory, and Ethical Frameworks for Implementation?

Before full implementation of AI in healthcare and LUTD care specifically, regulatory gaps need to be filled. The rapid development of AI, which vastly outpaces regulatory progress, is a major challenge. Governing bodies worldwide are developing strategies to cope with this, each with their own emphases and priorities. The EU has a risk‐based focus in the EU AI Act [9] and classifies AI used in clinical decision making under Medical Device Regulation (EMA/MDR) [10]. In contrast, the U.S. follows a more market‐oriented approach, where the FDA regulates AI in healthcare primarily under the framework of Software as a Medical Device (SaMD) [11], which emphasizes market entry and innovation rather than predefined risk tiers. There is consensus that key points of AI governance/regulation are: safety, respect for fundamental rights and values (first do no harm), promotion of trust in AI technologies and facilitation of innovation. A review by Palaniappan et al has formulated essential points for a coordinated legal, regulatory, and ethical framework for implementation of AI in healthcare [12]. The translation of these points to the application of AI in LUTD is discussed below.

The first is data quality, security and protection. Data should be representative and comply with the 4 V's: Volume, Variety, Veracity and Velocity. For LUTD, a uniform database structure for urodynamics data has been proposed [13], which may serve as the basis for an International Continence Society Data Repository [14]. Nevertheless, there are practical challenges. The use of data in AI algorithms needs to comply with data protection legislation such as the Health Insurance Portability and Accountability Act (HIPAA—US) or General Data Protection Regulation (GDPR—EU). One concern is the potential re‐identification by AI of patients from anonymized data, especially in rare diseases. Furthermore, previously provided informed consent may not include the permission to retrospectively use data for AI development. It may also be challenging to fully extract a patient's data from data‐based AI algorithms when the patient withdraws informed consent. Data repositories require data exportation across borders and jurisdictions, causing both legal and security concerns.

The second is validation of algorithms, which relates to transparency and trust. The output of AI should be explainable: explainability refers to the extent to which the internal reasoning of an AI model including the factors, features, and decision rules it relies on can be made understandable to a human audience. This is especially critical in LUTD, as symptoms can be subjective. A “black box” can lead to automation bias (over‐reliance on AI) or distrust, and will compromise shared decision‐making in healthcare [15]. Patient‐centered care is paramount in LUTD, not only because of the benign nature of these disorders and the preference for sensitive treatment options such as those available for OAB [16], but also due to the complex mind–body interplay that often underlies functional disorders like OAB [17]. Both clinicians and patients need to understand and be able to explain why diagnostic or therapeutic options are offered. The implication is that all AI healthcare applications should have in‐depth technical documentation including the purpose, procedure of development, comprehensive information on the data sets used for training and validation, similar to the information provided in clinical trials for medication or medical devices.

Another important consideration is accountability/liability. It is essential that both healthcare professionals and patients receive a specific disclosure when AI has been used in their diagnosis or treatment process. Examples are talking to chatbots or when accessing AI generated/assisted scientific content. AI should currently assist and not replace clinical judgment. Continuous learning models are a challenge in terms of liability: the product keeps developing after release (as opposed to locked models) and it may become unclear who is accountable for an error later in this process. Currently AI is included in the Product Liability Directive from the EU, and may be under Tort law in the US.

Last but not least is the ethics of and equitable access to AI. Real world patterns of health inequality and discrimination—for example, limited access to single‐use catheters, (ambulatory) urodynamics, nerve stimulation such as SNS, and robotic surgery, may translate into discriminatory data being used to train AI models. This will lead to both biased design and implementation practices, and hence application inequity of precision/personalized medicine [18]. Governance on non‐discriminative data used for training AI is therefore crucial. Finally, it is worth considering the climate impact of AI. Worldwide AI‐related electricity consumption may increase to 134 TWh annually, which is comparable to the annual electricity consumption of a small country [19].

## 4 What Standards and Levels of Evidence Are Needed to Demonstrate Value and Effectiveness of AI in LUTD?

With the growth of AI applications in healthcare, several standards have recently been developed. One example is the British Standards Institute having issued a validation framework for AI in healthcare in 2023. The framework includes ensuring the clinical effectiveness, external validity and equity and bias of the application [20, 21].

Levels of evidence in healthcare range from Level 1, which includes systematic reviews, individual RCTs and all or none situations, to Level 5, which includes expert opinions without explicit critical appraisal [22]. Where does AI belong? To answer this question, Probst and Wagner addressed levels of evidence in the era of AI [23]. AI may generate evidence faster and better, but must be reproducible and transparent. Chatbots that provide instant answers with no transparency or traceability would be at the lowest level of 5 or possibly at a proposed level 6 [23]. AI may efficiently generate systematic reviews with meta‐analyses of RCTs, and could provide level 1 evidence with appropriate documentation and sufficient human oversight and critical assessment [23].

Roles for AI in LUTD may include prediction, diagnosis and treatment decision making, as well as administration and improving patient safety, and the levels of evidence needed depend on the role. RCTs are the highest level of evidence for clinical interventions, and AI solutions for diagnosis/treatment should be assessed to the same standard. Effectiveness of AI solutions is essential, but value must also be demonstrated. Measures of effectiveness used for other studies, such as sensitivity, specificity and confidence intervals, should also be used for AI. Effectiveness may be more challenging to demonstrate if there is not a gold standard for comparison. Transparency and repeatability are critical, and there is potential for bias for or against the use of AI solutions. Potential risks of using AI in healthcare include false negatives in the form of missed diagnoses, unnecessary treatment due to false positives, unsuitable interventions due to imprecise diagnoses, or incorrect prioritization of interventions in emergency departments. Other potential risks include loss of doctor‐patient interaction and marginalization of vulnerable groups.

Experimental evidence does not necessarily equate to real‐world performance. AI may catch a diagnosis that a human missed, but a human may catch a diagnosis that AI missed [24]. Mechanisms for continuous monitoring and evaluation of AI system performance and efficacy are needed, along with improvement of AI systems based on monitoring and feedback.

Physicians need timely and trustworthy evidence because their actions may have immediate and potentially irreversibly consequences. AI cannot replace clinical trials or a physician”s judgement. AI may be effective if it can help the surgeon reach faster and more precise conclusions. To trust and effectively use AI, the physician may need to see the same data they would normally see and know the quality of the data compared to the data used for AI training.

## 5 What Are the Clinician and Patient Perspectives in Development and Deployment of AI Solutions?

The integration of AI into urology, especially functional urology, is rapidly advancing [25]. While much attention has centered on technological capabilities, less focus has been given to the perspectives of clinicians and patients, whose involvement is critical for successful adoption. From the clinician's viewpoint, AI offers the potential to enhance diagnostic accuracy, streamline workflows, and support complex decision‐making. In functional urology, AI has been applied in the interpretation of bladder diaries, uroflowmetry, filling cystometry, voiding pressure flow studies, and in predicting the outcomes of botulinum toxin and sacral nerve stimulation therapies [4, 26, 27, 28, 29, 30]. However, the performance of AI in these settings remains suboptimal, with AUC values ranging from 0.70 to 0.91, indicating the need for further development to improve accuracy. Clinician skepticism persists, often fueled by concerns regarding interpretability, accountability, and potential disruption to established workflows.

Additionally, the introduction of natural language models such as ChatGPT has broadened the potential applications of AI in functional urology. Recent evaluations of chatbot accuracy have yielded mixed results, ranging from highly accurate to entirely incorrect responses [31, 32, 33, 34, 35]. While recent advancements in natural language processing (NLP) have significantly improved performance, the “black box” phenomenon remains a key challenge. For AI solutions to achieve widespread adoption, transparency in algorithm design, rigorous validation, and seamless integration with existing clinical systems are essential.

Functional urology often relies on subjective assessments including bladder diaries, symptom scores, or patient‐reported outcomes, which are challenging to standardize. AI could facilitate automated data extraction and longitudinal monitoring, but clinicians need to trust the algorithm's outputs, especially when making decisions about invasive interventions like SNS or botulinum toxin injections. To address current limitations, AI and NLP tools must be adapted to diverse populations, cultures, and habits.

Moreover, the adoption of AI in healthcare faces significant financial barriers, including high upfront costs for infrastructure, data management, staff training, and ongoing maintenance. These challenges, particularly in low‐resource settings, may hinder integration despite potential long‐term benefits. This underscores the need for strategic investment and supportive policies to ensure equitable and sustainable implementation [36].

From the patient's perspective, the adoption of AI in urology raises concerns around data privacy, informed consent, and the potential depersonalization of care. Nevertheless, many patients are open to AI tools when they are presented as adjuncts to physician expertise rather than replacements [37]. In functional urology, where chronic symptoms can significantly impact quality of life, patients value human empathy and individualized care. Involving patients in the codesign of AI tools can improve their acceptability and usability [4, 26]. Mobile applications that combine symptom tracking with AI‐driven alerts or feedback may empower patients, but must be developed with attention to accessibility, health literacy, and data protection. In clinical practice, demands for increased transparency and explainability are particularly critical [36, 37]. Finally, from the patient's point of view, readability and understandability are of utmost importance and should always be considered.

## 6 Discussion and Conclusion

AI holds promise for transforming LUTD care, but its deployment must be guided by clinical need, data readiness, and adherence to legal and ethical frameworks. Conditions such as neurogenic LUTD, BOO, PUV, and OAB are clear opportunities wherein AI may optimize the diagnostic process, personalize treatment, and improve long term outcomes. AI in LUTD should be transparent, explainable, and validated with the same rigor as other clinical interventions. Equitable access will be necessary to avoid unintended harm or bias. Clinician‐led initiatives that bring together cross‐disciplinary teams—including engineers, ethicists, and patients—can ensure that AI applications address real clinical needs while preserving the humanistic core of urologic care. Future work should prioritize prospective studies that evaluate clinical utility, real‐world performance, and patient‐centered outcomes. Open and pressing research questions relating to the use of AI in LUTD, and strategies to address them, are included in Table 2.

## Ethics Statement

The authors have nothing to report.

## Consent

The authors have nothing to report.

## Conflicts of Interest

Glenn Werneburg: Consultant: Light Line Medical, Atterx Biotherapeutics, Clarametyx Biosciences; Medical Advisory Board: Renascent Diagnostics Michel Wyndaele: Consultant: Medtronic, Pierre Fabre, Coloplast – Invited Speaker: Wellspect, Hollister, Boston Scientific, Astellas Mo Belal: Consultant: Coloplast, Medtronic, – Invited Speaker: Abbvie, Astellas, Boston Scientific, Hollister, Medtronic, Pierre Fabre Wellspect George Bou Kheir: Invited Speaker: Wellspect, Travel grant: Astellas, Pierre Fabre, Ipsen Arjun Nambiar: Consultant: Medtronic; Invited speaker: Merck; Travel grant: Abbvie Phil Toozs‐Hobson: Funding support to attend conferences and present work Bluewind. The other authors declare no conflicts of interest.

## Data Availability

Data sharing not applicable to this article as no datasets were generated or analysed during the current study.
